# Long-term survival after intensive care for COVID-19: a nationwide cohort study of more than 8000 patients

**DOI:** 10.1186/s13613-023-01156-3

**Published:** 2023-08-29

**Authors:** Elsa Hägglöf, Max Bell, Erik Zettersten, Lars Engerström, Emma Larsson

**Affiliations:** 1 Department of Physiology and Pharmacology, Karolinska Institutet, Stockholm, Sweden; 2 Department of Perioperative Medicine and Intensive Care, Karolinska University Hospital, 171 76 Stockholm, Sweden; 3 Department of Anesthesiology and Intensive Care in Norrköping and Department of Medical and Health Sciences, Linköping University, Linköping, Sweden; 4 Department of Cardiothoracic and Surgery Anaesthesia and Department of Medical and Health Sciences, Linköping University, Linköping, Sweden; 5 The Swedish Intensive Care Registry, Linköping, Sweden

**Keywords:** COVID-19, Gender, Intensive care, Long-term outcome

## Abstract

**Background:**

Was it worth it—what is the outcome after the extended ICU (intensive care unit) length of stay for COVID-19 patients? Surprisingly, data on long-term mortality in large cohorts are lacking. We investigate long-term mortality including differences between men and women, as previous studies show that men generally suffer a more severe course of COVID-19 in terms of severity of illness and short-term mortality.

**Methods:**

Nationwide cohort including all adult COVID-19 patients admitted to Swedish ICUs until August 12, 2022. Primary outcome was 360-day mortality after ICU admission. Logistic regression was used to estimate associations between demographics, comorbidities, clinical characteristics and mortality.

**Results:**

In total, 8392 patients were included. Median (IQR) age was 63 (52–72) years and the majority (70.1%) were men. Among the 7390 patients with complete 360-day mortality data, 1775 (24.4%) patients died within 30 days, 2125 (28.8%) within 90 days and 2206 (29.8%) within 360 days from ICU admission. 360-day mortality was 27.1% in women and 31.0% in men. Multivariable logistic regression analysis showed increased risk of 360-day mortality in men compared to women (OR: 1.33, 95% CI: 1.17–1.52). Other variables associated with poor 360-day mortality were age, cardiac disease, COPD/asthma, diabetes, immune deficiency, chronic kidney disease, neuromuscular disease, and malignancy.

**Conclusion:**

This study confirms the increased severity of disease in critically ill men with COVID-19, even in a long-term perspective. However, mortality beyond 90 days was strikingly low, indicating high probability of survival after the acute phase of illness.

**Supplementary Information:**

The online version contains supplementary material available at 10.1186/s13613-023-01156-3.

## Background

The Coronavirus disease (COVID-19) pandemic has resulted in about 600 million cases, and more than 6.5 million global deaths has been reported [1]. The pandemic has had an enormous impact on health care, and critical care services worldwide were strained to their limits. Clinical characteristics of COVID-19 patients treated in the intensive care unit (ICU) are well known [2, 3] and short-term outcome has been extensively described in various settings [4, 5]. It is also well established that women generally suffer a less severe course of COVID-19 as compared to men; fewer women are hospitalized, admitted to ICUs and their short-term outcome is overall better [5].

Knowledge gaps remain. We lack data on long-term outcome in critically ill COVID-19 patients and risk factors associated with poor long-term survival. Specifically, what is the late mortality for ICU survivors and do men and women have similar mortality trajectories? In general, following discharge from critical care, most patients face a difficult recovery, and excess mortality has been reported after hospitalization [6]. If COVID-19 survivors have similar outcomes is unknown, as few large cohort studies focusing on long-term outcomes exist. To achieve comprehensive knowledge of long-term outcome after intensive care for COVID-19, analyses of population-based data with high validity and minimal loss to follow-up are necessary.

Accordingly, the present nationwide cohort study, based on all ICU admissions for COVID-19 in Sweden aimed to analyze long-term outcome after ICU admission, detailing risk factors for mortality including possible differences between men and women.

## Materials and methods

This study was approved by the Swedish Ethical Review Authority (approval number 2020-01477). All research was conducted in accordance with national guidelines and regulations. The study adhered to the STROBE (Strengthening the Reporting of Observational Studies in Epidemiology) guidelines for cohort studies [7].

As of September 8, 2022, 2,574,962 patients had been tested positive for SARS-CoV-2-RNA and 20,096 deaths were registered due to COVID-19 in Sweden [8]. During the study period just over 100,000 COVID-19 patients had been hospitalized in total [9].

### Study design and population

All public health care in Sweden, including intensive care, is tax funded and available for every citizen regardless of private health insurances. In this nationwide cohort study, we identified all ICU patients ≥ 18 years of age with confirmed SARS-CoV-2 by polymerase chain reaction admitted until August 12, 2022, in the Swedish Intensive Care Registry (SIR). SARS-CoV-2-RNA positive patients with other reason for admission than COVID-19 were excluded. That is, patients treated for symptoms unrelated to COVID-19, such as burns, intoxication and trauma but despite this had a positive test. Moreover, patients with a temporary or invalid Swedish personal identification number were also excluded since these patients are unavailable for follow-up. Patients were followed until death or study end-point September 8, 2022, whichever came first.

SIR is operative collecting individual patient data within the legal framework of the Swedish National Quality Registries [10]. Written informed consent is according to this framework not required, but all patients have the possibility to withdraw their data from the registry at any time. All 83 ICUs in Sweden are members of SIR. In co-operation with the Public Health Agency of Sweden, mandatory surveillance data of ICU patients with COVID-19 has routinely been reported to SIR during the entire pandemic. Available data in SIR include baseline demographics, comorbidities, variables included in the Simplified acute physiology score (SAPS3) and variables on treatment given within the ICU. After central validation at SIR, incomplete or inconsistent (entries outside pre-specified limits) patient data are returned to the individual ICUs for correction before data are added to the SIR database.

In Sweden, all citizens have a unique personal identity number assigned at birth or upon immigration to Sweden, enabling linkages between different national registries. Complete follow-up was ascertained by linkage between SIR and the Swedish population registry, where SIR receives information on all deceased or emigrated persons on a weekly basis. The personal identity number also enables analyses of readmissions and to follow the care of an individual patient treated at several ICUs.

### Covariates and outcomes

Patient demographics, characteristics and comorbidities were defined at the time of admission to ICU. Physiological variables were recorded within one hour before until one hour after ICU admission. The data with the most pathological values within this time interval were utilized as baseline parameters and to calculate SAPS3. The primary outcome was 360-day mortality, defined as all cause death within 360 days after admission to the ICU, and the secondary outcome was 90-day mortality.

### Statistical analysis

Categorical variables are presented as numbers with percentage, continuous variables are presented as median and interquartile range (IQR). Patients with less than 90-, 180- or 360-days follow-up time at the endpoint of the study were excluded from each respective mortality analysis. To compare mortality trajectories between female and male patients a survival analysis with Kaplan–Meier estimates was conducted, with comparison between curves using a log-rank test.

Associations with 360- and 90-day mortality and a priori selected variables including patient sex, age, comorbidities (cardiac disease, chronic obstructive pulmonary disease (COPD)/asthma, morbid obesity (BMI > 40 kg/m^2^), hypertension, immune deficiency, chronic liver disease, chronic kidney disease, neuromuscular disease and malignancy (neoplasia spread beyond regional lymph nodes)), simplified acute physiology score (SAPS3) and admission period (“wave 1” = March 6th to August 30th 2020; “wave 2” = 1st of September 2020 to 31st of January 2021; “wave 3” = 1st of February 2021 to 30th of November 2021; “wave 4” = 1st of December 2021 to 12th August 2022) were estimated using univariate and multivariable logistic regression models and expressed as odds ratios (OR) with corresponding 95% confidence intervals. Age and comorbidity components were removed from SAPS3 to avoid collinearity. All covariates in the univariate models were carried forward to the multivariable models. In addition, we performed a Cox regression model with the same covariates estimating factors associated with mortality.

A p-value of 0.05 was considered statistically significant. Stata/SE 16 (StataCorp, College Station, TX, USA) and R 4.1.1. R Core Team (2021. R: A language and environment for statistical computing. R Foundation for Statistical Computing, Vienna, Austria) were used for all data analyses.

## Results

### Patients

**Fig. 1:**
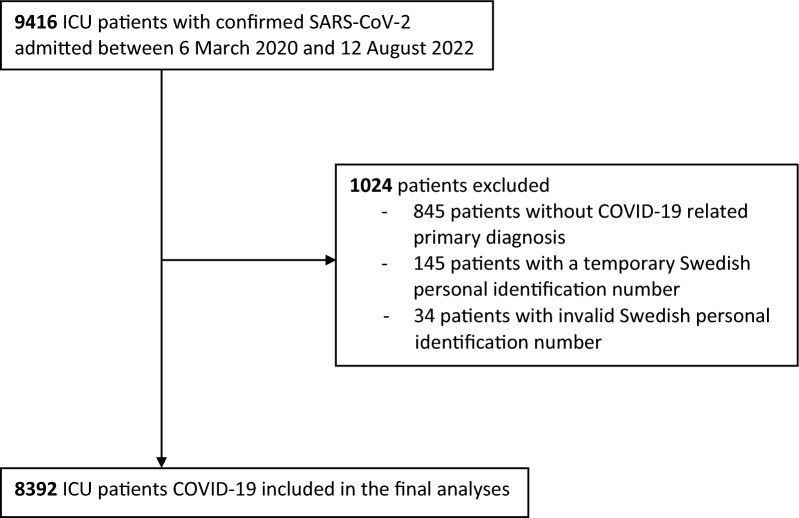
Flow chart of included patients

Until August 12, 2022, a total of 9416 ICU patients with confirmed SARS-CoV-2 were reported to SIR. We excluded 845 patients with a primary diagnosis not associated with COVID-19, 145 patients with a temporary identification number and thereby not possible to follow up as well as 34 patients with invalid registration of the Swedish personal identification number. In total, 8392 ICU patients with laboratory-confirmed COVID-19 were included in this study (Fig. 1).

### Demographics

Of the included 8392 patients, 2509 (29.9%) were women. Median age was 63 (IQR 52–72) years for the entire patient cohort, 63 (IQR 51–72) and 63 (IQR 54–72) years for women and men respectively. Roughly three quarters of the patients were admitted from hospital floor (76.4%). Most patients were first admitted to tertiary (33.8%) or county (51.8%) hospitals. Approximately one-third (30.1%) of the patients had no reported comorbidity at admission; 28.1% and 31.0% for women and men respectively. Morbid obesity, immune deficiency, COPD/asthma, chronic liver disease and neuromuscular disease were more commonly reported in women while chronic cardiac disease, chronic kidney disease, diabetes and malignancy were more common in men. Median SAPS3 score at admission was 55 (IQR 49–62), corresponding to a median predicted risk of death of 26% (IQR 14–40) and was identical for women and men. Patient demographics, characteristics, comorbidities, treatment and vital signs at ICU admission are presented in detail in Table 1.

**Table 1:** Baseline characteristics

	All	Women	Men	P-value
Patient demographics, characteristics and comorbidities at ICU admission				
No. (%)	8392	2509 (29.9)	5883 (70.1)	
Age, median (IQR), y	63 (53–72)	63 (51–72)	63 (54–72)	0.003
Age, Interval, y				
< 40	670 (8)	293 (11.7)	377 (6.4)	< 0.001
40–49	900 (10.7)	282 (11.2)	618 (10.5)	
50–59	1768 (21.1)	483 (19.3)	1285 (21.8)	
60–69	2392 (28.5)	651 (25.9)	1741 (29.6)	
70–79	2151 (25.6)	646 (25.7)	1505 (25.6)	
≥ 80	511 (6.1)	154 (6.1)	357 (6.1)	
Admission period^a^				
Wave 1	2428 (28.9)	649 (25.9)	1779 (30.2)	< 0.001
Wave 2	2104 (25.1)	617 (24.6)	1487 (25.3)	
Wave 3	3235 (38.5)	1021 (40.7)	2214 (37.6)	
Wave 4	625 (7.4)	222 (8.8)	403 (6.9)	
Location before ICU admission				
Emergency department	1981 (23.6)	548 (21.8)	1433 (24.4)	0.014
Hospital floor	6409 (76.4)	1960 (78.1)	4449 (75.6)	
Time from symptom to ICU admission, d				
Median (IQR)	10 (7–13)	10 (7–12)	10 (7–13)	< 0.001
Hospital level				
Tertiary	2840 (33.8)	815 (32.5)	2025 (34.4)	0.068
County	4349 (51.8)	1305 (52)	3044 (51.7)	
Local	1203 (14.3)	389 (15.5)	814 (13.8)	
Days at hospital before ICU admission, d				
No. with data	8284	2464	5820	
Median (IQR)	2 (0–4)	2 (0–4)	2 (0–4)	0.570
Pregnant, No. (%)	78 (0.9)	78 (3.1)	0 (0)	< 0.001
Comorbidities				
None	2527 (30.1)	705 (28.1)	1822 (31.0)	0.009
One or more	5865 (69.9)	1804 (71.9)	4061 (69.0)	0.009
Chronic hypertension	3717 (44.3)	1039 (41.4)	2678 (45.5)	0.001
Chronic cardiac disease	1363 (16.2)	299 (11.9)	1064 (18.1)	< 0.001
COPD/Asthma	1464 (17.4)	604 (24.1)	860 (14.6)	< 0.001
Immune deficiency	760 (9.1)	300 (12.0)	460 (7.8)	< 0.001
Chronic liver disease	79 (0.9)	29 (1.2)	50 (0.8)	0.228
Chronic kidney disease	526 (6.3)	152 (6.1)	374 (6.4)	0.640
Diabetes	2102 (25.0)	602 (24.0)	1500 (25.5)	0.153
Neuromuscular disease	162 (1.9)	61 (2.4)	101 (1.7)	0.037
Morbid obesity^b^	702 (8.4)	314 (12.5)	388 (6.6)	< 0.001
Malignancy^c^, No./total (%)	205/8284 (2.5)	59/2464 (2.4)	146/5820 (2.5)	0.819
Treatment and vital signs at ICU admission (within 1 h before until 1 h after admission)				
Vasopressor on admission, No./total (%)	353/8284 (4.3)	94/2464 (3.8)	259/5820 (4.5)	0.212
Fever^d^, No./total (%)	2386/7876 (30.3)	652/2340 (27.9)	1734/5536 (31.3)	0.002
Glasgow coma scale				
No. with data	3372	979	2393	
Median (IQR)	15 (14–15)	15 (14–15)	15 (14–15)	0.012
Systolic blood pressure, mmHg				
No. with data	8036	2382	5654	
Median (IQR)	120 (104–140)	120 (100–136)	120 (105–140)	< 0.001
Heart rate, beats/min				
No. with data	8117	2410	5707	
Median (IQR)	92 (80–109)	93 (80–110)	91 (80–108)	0.204
PaO_2_, kPa				
No. with data	7637	2267	5370	
Median (IQR)	8.8 (7.6–10.2)	8.7 (7.6–10.1)	8.8 (7.7–10.3)	0.090
FiO_2_, %				
No. with data	5723	1725	3998	
Median (IQR)	75 (60–90)	75 (60–90)	76 (60–90)	0.615
PaO_2_/FiO_2_ ratio, kPa				
No. with data	5672	1710	3962	
Median (IQR)	12.1 (9.3–16.2)	12 (9.2–16.17)	12.2 (9.4–16.3)	0.329
White blood cell count, × 10^9^/L				
No. with data	7622	2270	5652	
Median (IQR)	9 (6.5–12.4)	8.8 (6.3–12.4)	9.1 (6.7–12.3)	0.009
pH				
No. with data	7922	2352	5570	
Median (IQR)	7.45 (7.40–7.48)	7.44 (7.39–7.48)	7.45 (7.4–7.48)	< 0.001
Creatinine, mg/dL				
No. with data	7645	2272	5373	
Median (IQR)	72 (57–96)	58 (46–79)	77 (63–101)	< 0.001
Bilirubin, mg/dL				
No. with data	7393	2205	5188	
Median (IQR)	9 (6–12)	7 (5–10)	9 (7–13)	< 0.001
SAPS 3 at admission, median (IQR)				
No. with data	8284	2464	5820	
Median (IQR)	55 (49–62)	55 (49–62)	55 (49–62)	0.313
Predicted risk of death, median (IQR), %	0.26 (0.16–0.4)	0.26 (0.16–0.4)	0.26 (0.16–0.4)	0.313

*IQR* interquartile range, *y* years, *d* days, *ICU* intensive care unit, *COPD* chronic obstructive pulmonary disease, *PaO*_*2*_ arterial partial pressure of oxygen, *FiO*_*2*_ fraction of inspired oxygen, *SAPS* simplified acute physiology score

^a^Wave 1, 200306-200830; Wave 2, 200901-210131; Wave 3, 210201-211130; Wave 4, 211201-220812

^b^Morbid obesity is defined as a body mass index above 40 kg/m^2^

^c^Malignancy is defined as neoplasia spread beyond regional lymph nodes

^d^Body temperature above 38 °C

### Process of care

Overall, 63.5% of the patients received invasive mechanical ventilation for a median total duration of 10.9 (5.8–19.7) days. Renal replacement therapy and prone position were reported in 11.8% and 46.8% of the patients, respectively, and 1.3% were treated with extra corporeal membrane oxygenation (ECMO). Median total length of ICU stay was 8.2 (IQR 3.0–17.1) days. Care provided in the ICU for the entire cohort and for women and men separately are presented in Table 2.

**Table 2:** Care provided in the ICU

Variable	All (*n = *8392)	Women (*n = *2509)	Men (*n = *5883)	P-value
Highest level of respiratory support				
No. with data	8326	2495	5831	
Invasive mechanical ventilation, No. (%)	5288 (63.5)	1493 (59.8)	3795 (65.1)	< 0.001
Non-invasive mechanical ventilation, No. (%)	1371 (16.5)	448 (18)	923 (15.8)	
Duration of invasive mechanical ventilation				
No. with data	5288	1493	3795	
Median (IQR), d	10.9 (5.8–19.7)	9.5 (4.9–17.4)	11.6 (6.0–20.7)	< 0.001
Renal replacement therapy, No./total (%)	837/7121 (11.8)	183/2168 (8.4)	654/4953 (13.2)	< 0.001
Prone position, No./total (%)	3565/7625 (46.8)	963/2305 (41.8)	2602/5320 (48.9)	< 0.001
ECMO, No./total (%)	57/4446 (1.3)	16/1324 (1.2)	41/3122 (1.3)	0.890
Tracheostomy, No./total (%)	1842/8313 (22.2)	452/2492 (18.1)	1390/5821 (23.9)	< 0.001
One or more readmissions, No. (%), a	2133 (25.4)	573 (22.8)	1560 (26.5)	< 0.001
ICU length of stay, median (IQR), d	9.0 (3.6–18.0)	7.6 (2.8–15.1)	9.6 (3.9–19.0)	< 0.001
Outcome No/total (%)				
30-day mortality	2065/8392 (24.6)	581/2509 (23.2)	1484/5883 (25.2)	0.047
90-day mortality	2434/8329 (29.2)	671/2487 (27.0)	1763/5842 (30.2)	0.004
180-day mortality	2449/8239 (29.7)	668/2455 (27.2)	1781/5784 (30.8)	0.001
360-day mortality	2206/7390 (29.9)	590/2175 (27.1)	1616/5215 (31.0)	0.001

*IQR* interquartile range, *a* Includes readmission and transfers between ICUs, *d* days, *ECMO* extracorporeal membrane oxygenation, *ICU* intensive care unit

### Mortality

**Fig. 2:**
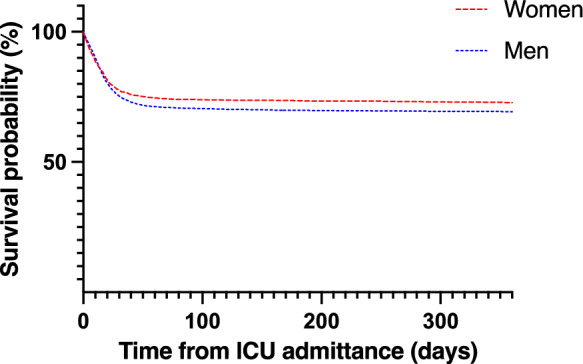
Kaplan–Meier survival plot after ICU admittance

Median follow-up time was 618 (IQR 520–837, range 41–929) days. Overall time to death is displayed in Fig. 2 (p = 0.03). Since 1002 patients in the cohort were admitted to the ICU less than 360 days before the endpoint of the study, complete 360-day follow-up data was available for 7390 patients. For the entire study cohort crude mortality at 90-days from ICU admission was 29.2%, in women 27.0% and in men 30.3%. The corresponding figures for mortality at 360 days were 29.9% for the entire study cohort, 27.1% and 31.0% for women and men respectively (Table 2). Among the 7390 patients with complete 360-day mortality data, 1775 (24.4%) patients died within 30 days from ICU admission, 2125 (28.8%) within 90 days, 2164 (29.2%) within 180 days and 2206 (29.8%) within 360 days from ICU admission. Patients treated with invasive ventilation had higher mortality rates at all times compared to not invasively ventilated patients (Table 3).

**Table 3:** Mortality in invasively ventilated patients compared to not invasively ventilated patients

Mortality No/total (%)	All (8392)	Invasive ventilation (5288)	No invasive ventilation (3038)	P-value
30-day mortality	2065/8392 (24.6)	1524 (28.8)	524 (17.2)	< 0.001
90-day mortality	2434/8329 (29.2)	1853 (35.2)	557 (18.6)	< 0.001
180-day mortality	2449/8239 (29.7)	1859 (35.7)	565 (19.1)	< 0.001
360-day mortality	2206/7390 (29.9)	1687 (35.7)	493 (19.0)	< 0.001

On univariate logistic regression analysis male sex, age, cardiac disease, COPD/asthma, diabetes, morbid obesity, hypertension, immune deficiency, chronic liver disease, chronic kidney disease, neuromuscular disease, malignancy, SAPS3 (excluding age and comorbidity components) and admission period were significantly associated with mortality. On multivariable logistic regression analysis, male sex (OR 1.33, 95% CI 1.17–1.52) remained significantly associated with mortality even after adjustment for the above-mentioned covariates. In addition, a majority of the other covariates remained significantly associated with 360-day mortality in the multivariable model (Table 4). We also performed a logistic regression model of 90-day mortality and a Cox regression model of overall mortality with almost identical results (Additional file 1: Table S1, S2).

**Table 4:** Univariate and multivariable logistic regression analysis for 360-day mortality (7322 patients included)

	Univariate		Multivariable^a^	
	OR (95% CI)	*P*-value	OR (95% CI)	P-value
Sex				
Women	Reference		Reference	
Men	1.21 (1.08–1.35)	0.0010	1.33 (1.17–1.52)	0.0000
Age, per year	1.08 (1.07–1.09)	0.0000	1.08 (1.07–1.09)	0.0000
Comorbidity				
Cardiac disease	2.64 (2.32–3)	0.0000	1.38 (1.19–1.6)	0.0000
COPD/Asthma	1.6 (1.41–1.82)	0.0000	1.51 (1.31–1.74)	0.0000
Diabetes	1.42 (1.27–1.59)	0.0000	1.17 (1.02–1.33)	0.0219
Morbid obesity^b^	0.71 (0.59–0.86)	0.0005	1.17 (0.93–1.45)	0.1734
Hypertension	1.63 (1.47–1.8)	0.0000	0.87 (0.77–0.98)	0.0274
Immune deficiency	1.9 (1.6–2.26)	0.0000	1.71 (1.4–2.09)	0.0000
Chronic liver disease	3.48 (2.12–5.8)	0.0000	2.01 (1.13–3.6)	0.0176
Chronic kidney disease	2.19 (1.8–2.66)	0.0000	1.14 (0.91–1.42)	0.2529
Neuromuscular disease	1.81 (1.2–2.71)	0.0041	2.06 (1.25–3.39)	0.0044
Malignancy^c^	2.99 (2.2–4.07)	0.0000	1.52 (1.08–2.15)	0.0157
SAPS3, per 1 unit increase^d^	1.07 (1.06–1.07)	0.0000	1.06 (1.05–1.07)	0.0000
Admission period^e^				
Wave 1	Reference		Reference	
Wave 2	1.34 (1.18–1.52)	0.0000	0.85 (0.74–0.99)	0.0000
Wave 3	0.98 (0.87–1.11)	0.8013	0.84 (0.74–0.97)	0.0142

*OR* odds ratio, *CI* confidence interval, *COPD* chronic obstructive pulmonary disease, *SAPS* simplified acute physiology score

^a^7322 patients included in the univariate and multivariable models

^b^Defined as BMI > 40 kg/m^2^

^c^Malignancy is defined as neoplasia spread beyond regional lymph nodes

^d^Recalculated after excluding age and comorbidities

^e^Wave 1, 200306-200830; Wave 2, 200901-210131; Wave 3, 210201-211130; Wave 4, 211201-220812

## Discussion

This national cohort study of 8392 patients includes virtually all ICU patients with COVID-19 in Sweden, with complete 360-day follow-up in 7390 patients. The mortality beyond 90 days was strikingly low, indicating high probability of survival after the acute phase of illness. Male patients had an increased risk of mortality compared to female patients and these differences remained after adjustment for relevant confounders.

To our knowledge this is the largest study presenting one-year mortality in COVID-19 patients treated in the ICU. Previous studies regarding long-term outcome in ICU-treated COVID-19 patients are few, with large variations in follow-up time and include relatively few patients. In a prospective study from California of 275 patients admitted to the ICU for COVID-19, 74.5% were discharged alive [11]. A systematic review of mortality rates in the ICU was published in June 2020 [12]. After exclusions this report included 15 full text studies from seven countries, and the ICU mortality was 25.7%, with the important caveat that 56.1% of patients were still in the ICU at the time of study publication: calculation of mortality based on a sample of only deceased or discharged patients risk painting a skewing reality. A Spanish multicenter investigation of 868 invasively ventilated patients with COVID-19 revealed a 180-day mortality of 41%, in contrast to the 29% mortality in this cohort with 63.5% invasively ventilated patients, despite almost identical median age and SAPS3 at admission [13]. Our results among patients who were invasively ventilated showed more comparable mortality rates, 35.7% at 180 days, indicating an increased mortality compared to not invasively ventilated but still lower rates than other studies. In Argentina, a study of 1909 invasively ventilated patients with COVID-19, with a median age of 62 years, was performed [14]. ICU and in-hospital mortality was 57.0 and 57.7%, respectively. Among 116 patients admitted to an ICU and treated with invasive ventilation in Italy, 47.4% died in hospital while no patient at 1-year follow up had died after discharge [15]. A Brazilian study including 428 patients, of whom 61% were treated with invasive ventilation, found a 12-month mortality of only 4% after surviving ICU-treatment [16]. A Danish study with 323 patients admitted to the ICU, where 82% were treated with invasive ventilation, showed an in-hospital mortality of 33.5% [17] and a follow-up study reported mortality at 6- and 12-months (37% and 38%, respectively) [18] suggesting that the majority of the mortality occurs at an early stage. This is consistent with our results in both the invasively ventilated group and the non-invasively ventilated, where mortality appears to decelerate after 90 days.

A comprehensive analysis of outcome after critical illness includes several dimensions, ranging from comorbidities to quality of life. In a study including 85 ICU-treated COVID-19 patients, 96.3% had abnormal chest CT scans after 12 months and 11% showed a lung diffusion capacity below normal lower limit [19]. A Dutch cohort of 246 patients alive after one year following COVID-19 and ICU-care report that 74.5% of the patients have remaining physical symptoms, 26.2% have mental symptoms, but less than 16.2% report cognitive symptoms [20]. It is obvious that the ICU research field must consider reporting outcomes beyond mortality, as indicated by a recent study of more than 77,000 ICU survivors [21].

During the pandemic it has become apparent that male patients are hospitalized and ICU-treated for COVID-19 to a higher extent as compared to female patients, and their short-term ICU-outcomes are worse [5, 22]. Our study appears to be the first to confirm this association even at 360-days of follow-up. Underlying potentially explaining factors to alterations between men and women in the context of COVID-19 have been studied during the pandemic. Consensus seems to exist that the variances are multifactorial where no single process serves as a comprehensive explanatory model. Differences between men and women could partly be understood by differences in the immune system. Sex hormones are active in regulating the immune system [23]. Specific parts of the immune response that have been suggested to play a role in COVID-19 include the pattern recognition receptor TLR7, which is X-linked and more expressed in females [24], the protein ACE2 which has been described as the entry pathway of the SARS-CoV-2 [25] and differences in antibody responses which could lead to immune-mediated pathology in men [26]. In fact, it has been suggested that estrogen supplementation is associated with decreased risk of COVID-19 mortality [27]. Gender differences, based on societal norms, may also affect admission rates, treatment and outcomes.

### Strengths and limitations

We analyzed a large, nationwide multicenter cohort of ICU patients with COVID-19 with almost complete follow-up. All COVID-19 patients cared for in an ICU in Sweden were identified in the study, enabling high generalizability to similar health-care systems. SIR includes high-resolution data including pre-ICU comorbidities, process of ICU care and long-term follow-up. Moreover, data are prospectively reported for quality-surveillance purposes and as such, unbiased in relation to the aim of this study. The length of the study period was two years, thereby including several waves of the pandemic.

This investigation has limitations inherent in most observational and registry-based studies within the field. First, we had no data on socioeconomic status and ethnicity. Moreover, we did not have data on ICU staffing as ICU resources were without a doubt strained during parts of the study period. Since previous studies indicate that drug treatment during intensive care for COVID-19 affects long-term mortality [28], data on drug treatment would have added value to the article. The study is also limited using mortality as only outcome variable, data on quality of life or days alive and at home would have been desirable. However, the relatively long study period in the context of COVID-19 studies must be regarded as a major strength by enabling adjustment for admission period. In addition, the worldwide cumulative knowledge concerning treatment of the disease continuously increased during the entire course of the pandemic, and treatment algorithms are constantly evolving.

## Conclusion

This study confirms the previously reported increased severity of disease in men compared to women in critically ill patients with COVID-19. In addition, our findings are paramount for policy makers in the health care sector, where the overarching question has lingered: what is the outcome after the extended ICU length of stay of COVID-19 patients? COVID-19 patients do have longer length of stay than patients with influenza [29] and indeed as compared to the average Swedish ICU patients, where the Swedish intensive care registry report that median and mean ICU length of stay was 2.66 and 1.07 days, respectively [30]. Despite this, and despite the high number of patients with high illness severity and comorbid burden, their long-term outcome, where seven out of 10 patients survive during a full year of follow-up, must be seen as a long-term benefit. In countries without national personal identification numbers, where long-term follow-up is difficult, costly and generally hard to assess—this study clearly indicates that 90-day mortality captures most deaths.

### Supplementary Information

**Additional file 1: Table S1.** Univariate and multivariable logistic regression analysis for 90-day mortality (8223 patients included). **Table S2.** Cox regression analysis for mortality (8284 patients included).

## Data Availability

The datasets used during the current study are available from the corresponding author on reasonable request.

## Abbreviations

COPD: Chronic obstructive pulmonary disease
COVID-19: Coronavirus disease 2019
ICU: Intensive care unit
IQR: Interquartile range
OR: Odds ratio
SAPS3: Simplified acute physiology score 3
SIR: Svenska intensivvårdsregistret (Swedish Intensive Care Registry)
STROBE: Strengthening the Reporting of Observational Studies in Epidemiology
