# Supplementary material for: Long-term survival after intensive care for COVID-19: a nationwide cohort study of more than 8000 patients
Source: Ann Intensive Care. 2023 Aug 29;13:76. doi: 10.1186/s13613-023-01156-3 (PMC10465451; doi:10.1186/s13613-023-01156-3)
Supplement: Supplementary file 1 — Additional file 1: Table S1. Univariate and multivariable logistic regression analysis for 90-day mortality (8223 patients included). Table S2. Cox regression analysis for mortality (8284 patients included). [file 13613_2023_1156_MOESM1_ESM.docx]

**Additional file 1: Table S1. Univariate and multivariable logistic regression analysis for 90-day mortality (8223 patients included)**

|  | Univariate | | Multivariable^a^ | |
| --- | --- | --- | --- | --- |
|  | OR (95% CI) | P value | OR (95% CI) | P value |
| Sex |  |  |  |  |
| Women | Reference |  | Reference |  |
| Men | 1.17 (1.05 - 1.3) | 0.0033 | 1.27 (1.12 - 1.43) | 0.0001 |
| Age, per year | 1.07 (1.07 - 1.08) | 0.0000 | 1.08 (1.07 - 1.08) | 0.0000 |
| Comorbidity |  |  |  |  |
| Cardiac disease | 2.5 (2.22 - 2.82) | 0.0000 | 1.32 (1.15 - 1.51) | 0.0001 |
| COPD/Asthma | 1.6 (1.42 - 1.8) | 0.0000 | 1.43 (1.25 - 1.64) | 0.0000 |
| Diabetes | 1.36 (1.22 - 1.51) | 0.0000 | 1.12 (0.99 - 1.27) | 0.0780 |
| Morbid obesity^b^ | 0.73 (0.61 - 0.87) | 0.0006 | 1.17 (0.95 - 1.44) | 0.1376 |
| Hypertension | 1.58 (1.43 - 1.73) | 0.0000 | 0.87 (0.78 - 0.98) | 0.0190 |
| Immune deficiency | 1.92 (1.64 - 2.24) | 0.0000 | 1.71 (1.43 - 2.05) | 0.0000 |
| Chronic liver disease | 2.71 (1.73 - 4.26) | 0.0000 | 1.47 (0.87 - 2.46) | 0.1461 |
| Chronic kidney disease | 2.14 (1.79 - 2.57) | 0.0000 | 1.11 (0.9 - 1.36) | 0.3196 |
| Neuromuscular disease | 1.62 (1.16 - 2.24) | 0.0038 | 2.14 (1.42 - 3.22) | 0.0003 |
| Malignancy^c^ | 2.49 (1.88 - 3.3) | 0.0000 | 1.34 (0.98 - 1.83) | 0.0671 |
| SAPS3, per 1 unit increase^d^ | 1.07 (1.06 - 1.07) | 0.0000 | 1.06 (1.06 - 1.07) | 0.0000 |
| Admission period^e^ |  |  |  |  |
| Wave 1 | Reference |  | Reference |  |
| Wave 2 | 1.31 (1.15 -1.49) | 0.0000 | 0.85 (0.73 – 0.98) | 0.0272 |
| Wave 3 | 0.97 (0.86 – 1.09) | 0.5736 | 0.83 (0.72 – 0.94) | 0.0052 |
| Wave 4 | 1.62 (1.33 – 1.96) | 0.0000 | 0.86 (0.69 – 1.08) | 0.2062 |

Abbreviations: OR, odds ratio; CI, confidence interval; COPD, chronic obstructive pulmonary disease; SAPS, simplified acute physiology score

^a^8223 patients included in the univariate and multivariable models

^b^Defined as BMI >40kg/m^2^

^c^Malignancy is defined as neoplasia spread beyond regional lymph nodes

^d^Recalculated after excluding age and comorbidities

^e^Wave 1, 200306-200830; Wave 2, 200901-210131; Wave 3, 210201-211130; Wave 4, 211201-220812

**Additional file 1: Table S2. Cox regression analysis for mortality (8284 patients included)**

|  | Univariate | | Multivariable^a^ | |
| --- | --- | --- | --- | --- |
|  | OR (95% CI) | P value | OR (95% CI) | P value |
| Sex |  |  |  |  |
| Women | Reference |  | Reference |  |
| Men | 1.1 (1.01 - 1.2) | 0.0289 | 1.13 (1.04 - 1.24) | 0.0060 |
| Age, per year | 1.06 (1.06 - 1.07) | 0.0000 | 1.06 (1.06 - 1.07) | 0.0000 |
| Comorbidity |  |  |  |  |
| Cardiac disease | 2.21 (2.02 - 2.41) | 0.0000 | 1.26 (1.15 - 1.38) | 0.0000 |
| COPD/Asthma | 1.5 (1.37 - 1.65) | 0.0000 | 1.36 (1.24 - 1.5) | 0.0000 |
| Diabetes | 1.32 (1.21 - 1.44) | 0.0000 | 1.11 (1.02 - 1.22) | 0.0168 |
| Morbid obesity^b^ | 0.75 (0.64 - 0.88) | 0.0003 | 1.07 (0.91 - 1.25) | 0.4111 |
| Hypertension | 1.48 (1.37 - 1.6) | 0.0000 | 0.9 (0.83 - 0.98) | 0.0108 |
| Immune deficiency | 1.83 (1.64 - 2.05) | 0.0000 | 1.62 (1.44 - 1.82) | 0.0000 |
| Chronic liver disease | 2.32 (1.72 - 3.13) | 0.0000 | 1.25 (0.92 - 1.69) | 0.1519 |
| Chronic kidney disease | 2.01 (1.77 - 2.29) | 0.0000 | 1.1 (0.97 - 1.26) | 0.1469 |
| Neuromuscular disease | 1.69 (1.33 - 2.13) | 0.0000 | 1.81 (1.42 - 2.31) | 0.0000 |
| Malignancy^c^ | 2.43 (2.03 - 2.92) | 0.0000 | 1.37 (1.14 - 1.66) | 0.0009 |
| SAPS3, per 1 unit increase^d^ | 1.06 (1.05 - 1.06) | 0.0000 | 1.05 (1.04 - 1.05) | 0.0000 |
| Admission period^e^ |  |  |  |  |
| Wave 1 | Reference |  | Reference |  |
| Wave 2 | 1.29 (1.16 - 1.43) | 0.0000 | 0.9 (0.81 - 1) | 0.0467 |
| Wave 3 | 0.98 (0.88 - 1.08) | 0.6508 | 0.85 (0.77 - 0.94) | 0.0021 |
| Wave 4 | 1.64 (1.42 - 1.9) | 0.0000 | 0.85 (0.73 - 0.99) | 0.0319 |

Abbreviations: HR, hazard ratio; CI, confidence interval; COPD, chronic obstructive pulmonary disease; SAPS, simplified acute physiology score

^a^8284 patients included in the univariate and multivariable models

^b^Defined as BMI >40kg/m^2^

^c^Malignancy is defined as neoplasia spread beyond regional lymph nodes

^d^Recalculated after excluding age and comorbidities

^e^Wave 1, 200306-200830; Wave 2, 200901-210131; Wave 3, 210201-211130; Wave 4, 211201-220812
